# Expression profiling of genes coding for abundant proteins in the alkenone body of marine haptophyte alga *Tisochrysis lutea*

**DOI:** 10.1186/s12866-019-1430-x

**Published:** 2019-03-11

**Authors:** Qing Shi

**Affiliations:** 1 School of Scientific Research, China University of Geosciences (Beijing), 29 Xueyuan Road, Beijing, 100083 China; 2 State Key Laboratory of Biological and Environmental Geology, China University of Geosciences (Beijing), Beijing, China

**Keywords:** Biofuel, Alkenone body, Haptophyte, Lipid body, Gene expression

## Abstract

**Background:**

Several abundant proteins have been identified in lipid body of an alkenone-producing marine haptophyte alga *Tisochrysis lutea.* The gene expression patterns of these proteins were investigated to better understand their roles in alkenone biosynthesis. For this purpose, *T. lutea* was first cultured in nitrogen-sufficient medium for biomass production and then shifted to nitrogen-deprived medium to induce lipid body formation.

**Results:**

There were remarkable increases in the volume of alkenone body (AB) and alkenone content in the alga after they were exposed to nitrogen depletion medium. Relative mRNA levels of the genes coding for the identified proteins V-ATPase subunit V_A_, V-ATPase subunit V_d_, hypothetical protein EMIHUDRAFT_465,517, coccolith scale associated protein-1, cycloartenol-c-24-methyltransferase 1-like and SPFH domain-containing protein were investigated over the culture period. RT-PCR data showed that the expression of all these genes except the gene coding for SPFH domain-containing protein was up-regulated during the transition period from nitrogen-sufficient to nitrogen-deficient medium. Among them, the expression of the coccolith scale associated protein-1 gene was up-regulated 50–650 folds. These up-regulations were consistent with the increased alkenone production in nitrogen-deprived medium, suggesting that these proteins are involved in alkenone biosynthesis in *T. lutea.*

**Conclusions:**

Expression analysis of the lipoprotein genes suggests that five out of the six genes are up-regulated and are therefore likely to code for the identified lipoproteins associated with alkenone biosynthesis in *T. lutea*. These data would help better understand alkenone metabolism and engineer for improved biofuel production in *T. lutea.*

## Background

Oil/lipid generated by microalgae is an attractive source of biofuel. Some species of alga are known to produce oil of up to 70% of their dry weight under optimal growth conditions and can produce about 20 times or more materials used for biodiesel production per unit area than the best oil-seed crops do [1, 2]. What is more, algae can be grown in marginal areas such as arid lands or potentially the ocean, and need less agricultural land and fresh water compared with food crops.

Microalgae are a group of unicellular or simple multicellular photosynthetic microorganisms that fix CO_2_ efficiently from different sources, including the atmosphere, industrial exhausted gases, and soluble carbonate salts. For these reasons, microalgae have attracted increasing attention and are being exploited as a renewable source of oil [3, 4].

Marine haptophyte algae are one of the greatest producers of biomass in the oceans [5]. Haptophytes contain calcifying species, coccolithophores, and non-calcifying species. Non-calcareous haptophyte species *Tisochrysis lutea,* previously named as *Isochrysis* aff. *galbana* (Clone Tahiti), is a taxonomic variation of *Isochrysis* species [6], and has been well-studied in aquaculture research for its mass cultivation as a commercial feed for fish [7, 8]. Lipids of *T. lutea* are commercially used as a nutrition source for the larvae due to their high content of long-chain polyunsaturated fatty acids such as docosahexaenoic acid (DHA) [9, 10]. *T. lutea* is genetically distinct from *I. galbana* [11], and biochemically, there are also some differences like their lipid content in sterol [12] and DHA [10]. *T. lutea* grows very fast over a broader broader temperatures range than *I. galbana* does [13].

Some species of Isochrysidales produce various lipid molecules of long-chain ketones, called alkenones [14–16], but not glycerolipids such as triacylglycerol (TAG). Different alkenones have been identified in *T. lutea* [16] and these molecules have a carbon chain length between C_37_ and C_40_ and carry two to four *trans*-type double bonds and one *keto*-group [17]. So far, only five strains of haptophytes in the order Isochrysidales are known to produce alkenones [16, 18]. Previous studies demonstrate that these alkenones are stored in lipid bodies (LBs) and function as a storage lipid in the cells [10, 19]. However, the cellular machinery, metabolic process and molecular mechanisms for their synthesis remain largely unknown [20–22].

Our proteomic analyses have revealed that there are several abundant proteins in the alkenone bodies (ABs) of *T. lutea* [23]. However, it is unclear if these proteins are AB-specific and whether they are related to alkenone production. To better understand the role of these proteins, we set to investigate the expression of these genes at mRNA levels with regards to alkenone production during cell growth and AB accumulation since they are most abundant. In the early study, although these proteins were identified but were not characterized for the roles and functions. Since these proteins, such as V-ATPase are biologically important, we set to profile the expression of these genes over the cell growth period with regarding to lipid synthesis to investigate their biological functions. Since transgenic technique is not available for the alga, cellular localization of these proteins using GFP is not possible and expression profiling remains a choice to associate these genes with lipid synthesis.

## Results

### Algal growth and AB formation

**Fig. 1:**
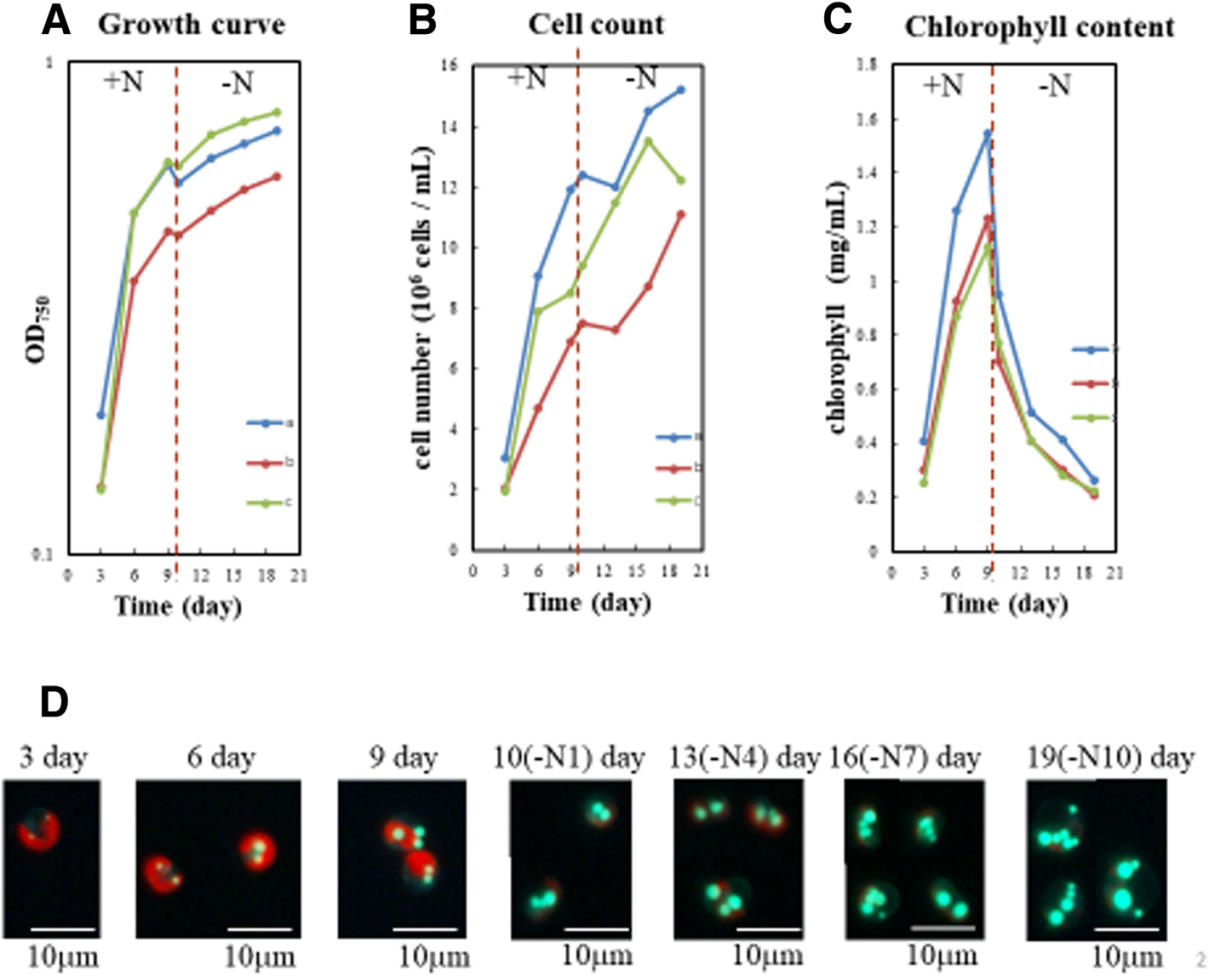
Cell growth curve, chlorophyll content and AB formation in the batch culture of haptophyte *T. lutea*. **a** Cell density in batches **a**, **b**, and **c**. **b** Cell number in batches **a**, **b**, and **c**. **c** Chlorophyll content in batches **a**, **b**, and **c**. **d** AB images during cell growth. Cells were stained with BODIPY 493/503. +N, nitrogen-sufficient medium. –N, nitrogen-deficient medium

*T. lutea* cells grew exponentially in N-sufficient medium containing 1.4 mM nitrate in a batch culture for 9 days (Fig. 1a and b). However, after shifted to nitrogen-deprived medium on day 9, their growth entered an early stationary phase, ABs were first observed as small BODIPY-stained neutral lipid particles, which become larger in size and more in number at the stationary growth phases after 10 days (Fig. 1d). During this period, chlorophyll content declined quickly (Fig. 1c). At the stage, the ABs remained as individual entities. However, some of them appeared as large ABs under the microscope due to image overlapping (Fig. 1d). This feature is different from the LBs produced in the unicellular green alga *Chlorella* where large-sized ABs are formed due to the fusion of several ABs within a cell [24]. The LBs of *Chlorella* mainly contain TAG, but the lipids in *T. lutea* cells have high content of alkenones [9, 10, 14]. These differences might be responsible for observed difference in AB formation.

### Alkenone and FAME synthesis

**Fig. 2:**
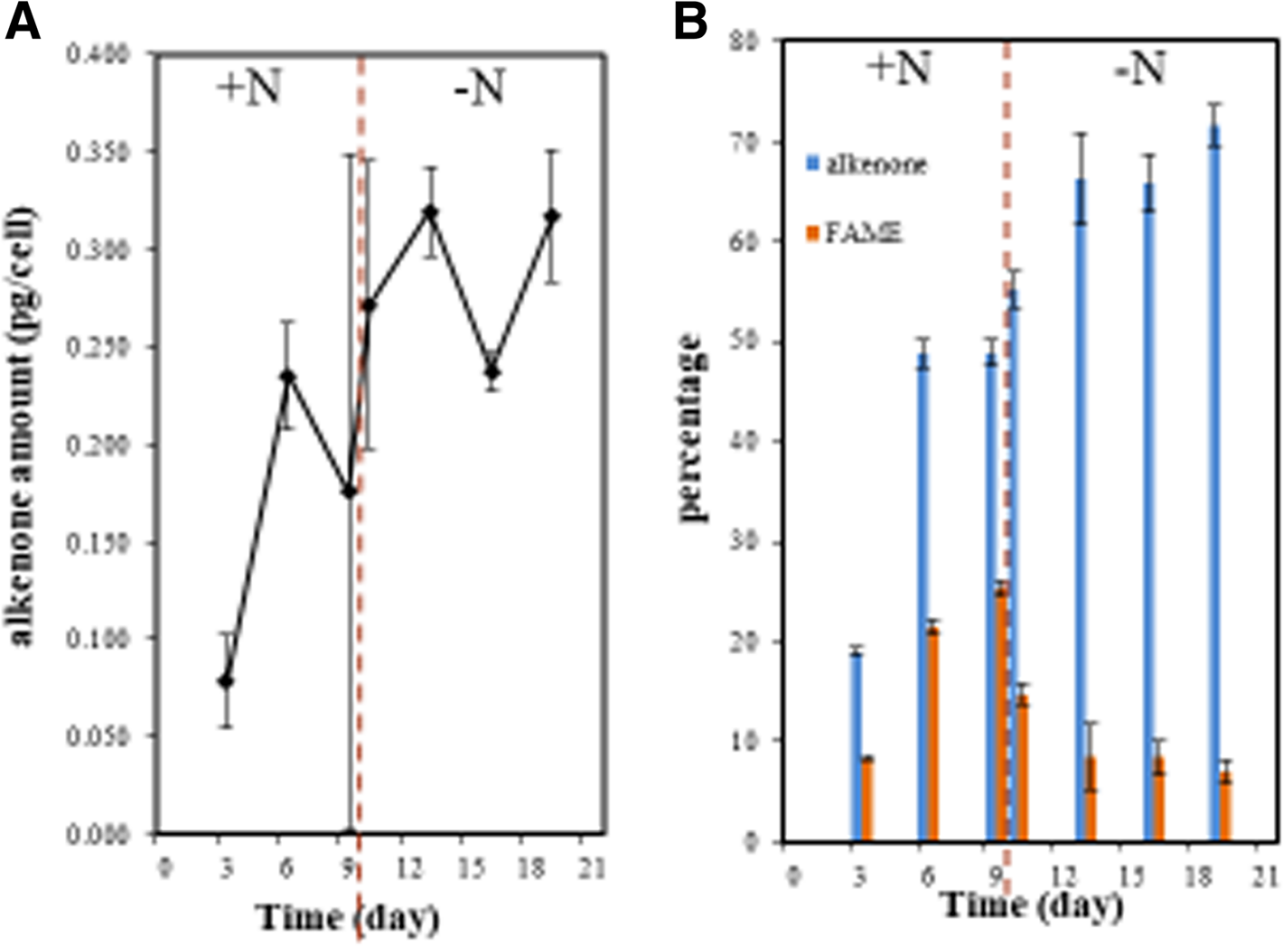
Alkenone and FAME synthesis during nitrogen-sufficient and –deficient periods. **a**. Alkenone content, **b**. Percentage of alkenone and FAME. +N, nitrogen-sufficient medium. –N, nitrogen-deficient medium

During the culture periods, the alkenone content and percentage continuned to increase as the culture time increased, while the percentage of fatty acid methyl ester (FAME) decreased after the cells were transferred to nitrogen-deption medium (Fig. 2). From the logarithmic phase to stationary phase, the proportion of alkenone and FAMEs had a leap rise, but after the cell was transferred into the nitrogen-deficient medium, the percentages of the two lipids over the total liqids were maintained basically unchanged at 75%.

### Gene expression

**Fig. 3:**
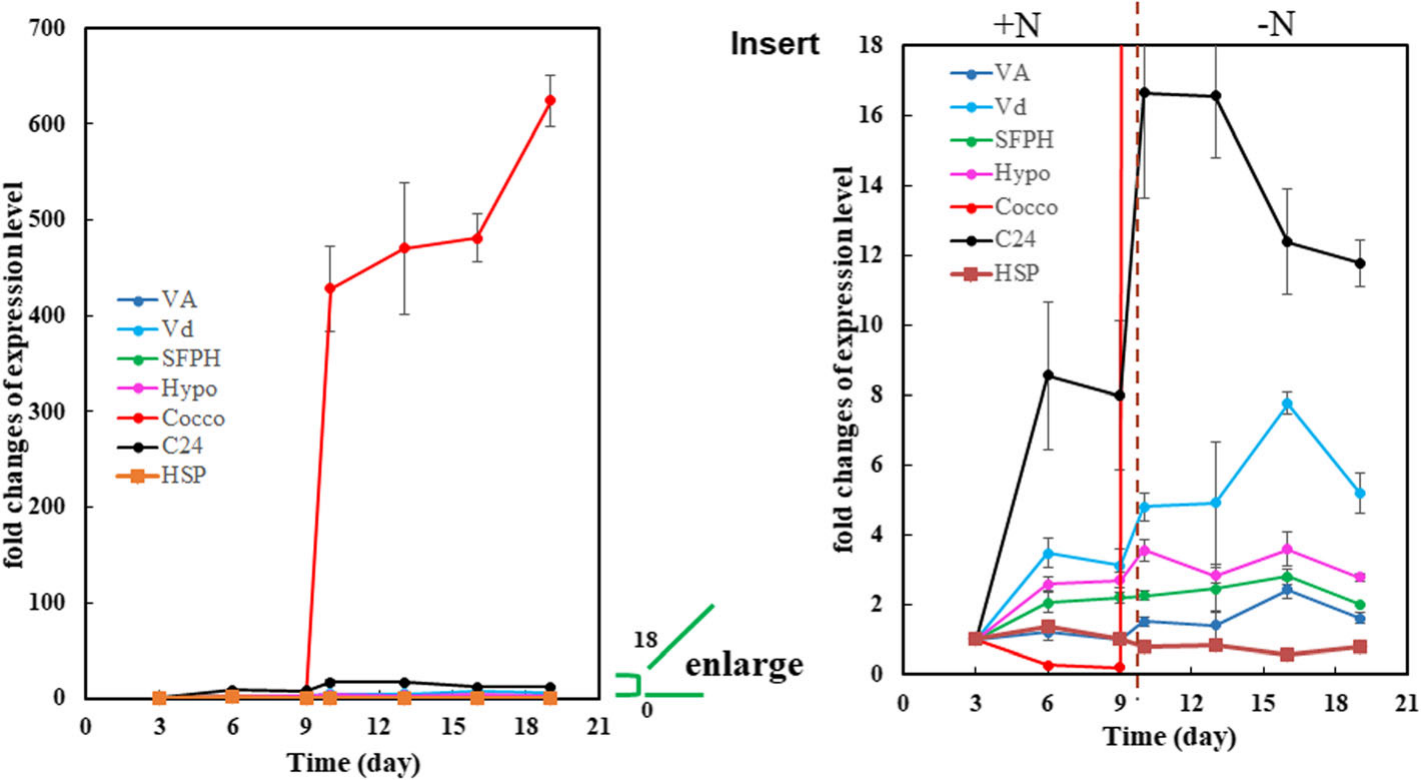
Relative mRNA levels of genes coding for abundant AB-associated protein during nitrogen-sufficient and –deficient periods. +N, nitrogen-sufficient medium. –N, nitrogen-deficient medium. Insert: enlarged part of Fig. 3. Va: V-type H+ ATPase complex V1 subunit A. Vd: V-type H+ ATPase complex V0 complex subunit d. Hypo: hypothetical protein EMIHUDRAFT_465,517. Cocco: coccolith scale associated protein-1. C24: cycloartenol-c-24-methyltransferase 1-like. SFPH: SFPH domain-containing protein

Proteomics data show that there are several abundant proteins in LPs [23]. However, their roles have not been characterized. To examine the expression of the genes coding for the proteins, we profiled their expression over the cell culture periods, especially during the transition period from nutrient-sufficient to nitrogen depletion conditions (from day 9 to 21), when there was a remarkable increase in AB size (Fig. 1d). PCR analysis showed that the mRNA levels of genes coding for the six major proteins were at different levels (Fig. 3a and b). Among them, V-ATPase domain V_0_, V-ATPase domain V_1,_ hypothetical protein EMIHUDRAFT_465,517, coccolith scale associated protein-1 and cycloartenol-c-24-methyltransferase 1-like were obviously up-regulated when the cells were transferred to nitrogen depletion condition, especially coccolith scale associated protein-1, those expression increased over 500 times. On other hand, the mRNA level of SPFH domain-containing protein remained unchanged. In addition, expression of some of these genes such as Cocco, V-ATPase domain V_A_ and V_d_ began to increase during the stationary phase when alkenone began to accumulate before the cells were transfer to N-deficient medium (days 6–9, Fig. 2), While others remains relatively stable before the transition.

## Discussion

**Fig. 4:**
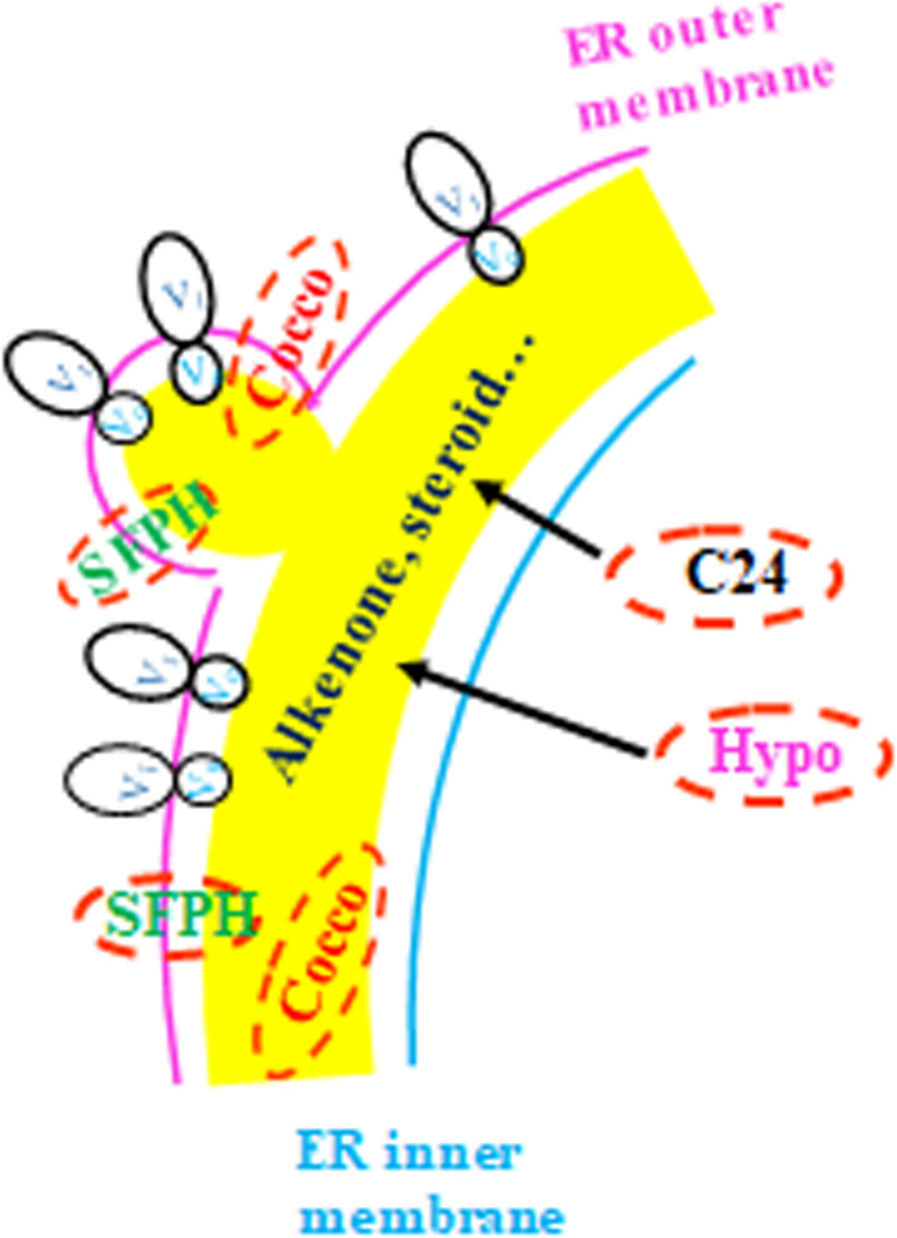
Proposed locations of AB-associated proteins in *T. lutea.* VA: V-type H+ ATPase complex V1 subunit A. Vd: V-type H+ ATPase complex V0 complex subunit d. Hypo: hypothetical protein EMIHUDRAFT_465,517. Cocco: coccolith scale associated protein-1. C24: cycloartenol-c-24-methyltransferase 1-like. SFPH: SFPH domain-containing protein. ER: endoplasmic reticulum

Our earlier proteomic study identified over 514 protein sequences and among them, the top 18 abundant proteins have hypothetically annotated functions as such proton pumps (V-ATPase), cytoskeletons, transferases and energy metabolisms [23] Since in the alga alkenones have been shown to be the major neutral lipids [16], it is of great interest to study the relationship between these proteins and alkenone synthesis. This study showed that there were remarkable increases in AB volume and alkenone content in the alga after it was exposed to nitrogen depletion medium. In parallel, five out of the six genes coding for AB-associated proteins V-ATPase subunit V_A_ (Va), V-ATPase subunit V_d_ (Vd), hypothetical protein EMIHUDRAFT_465,517 (Hypo), coccolith scale associated protein-1 (Cocco) and cycloartenol-c-24-methyltransferase 1-like were up-regulated, suggesting that these genes are involved in AB development and alkenone synthesis. Lipids in plant and alga are synthesized in ER [25]. Since alkenones are the major components of lipids in *T. lutea,* they are likely synthesized in ER as well. As such, the proteins identified with AB may be located in ER (Fig. 4), although no direct cellular localization has been established due to the lack of transgenic study.

V-ATPase (Vacuolar-type H^+^-ATPase) has two domains V_0_ (consisting of subunits such as Va, Vb, Vc and Ve and V_1_ (including subunits such as A-H) [26]. The expression level of the domain V_1_ in this study is virtually similar to that in a previous report on whole cell proteomic analysis of nitrogen-deprived *I.galbana* cells [27], where the gene was up-regulated around two to three folds under nitrogen depletion condition. In other hand, the expression of the V-ATPase domain V_0_ has not been reported previously. In the present study, the expression level of V-ATPase domain V0 was up-regulated 5–8 folds, which is much greater than that of domain V_1._ According to a previous study [28], the V_0_ domain is likely participated in membrane fusion since it is extremely hydrophobic, which may help lipid bilayer mixing after tethering of vesicles. V-ATPase domain V_0_ is known to be assembled in ER [29]. Therefore, the domain V_0_ of *T. lutea* may be localized in ER-derived membranes. From all these evidences, it is likely that V-ATPase involved in AB is located on the membrane that surrounds AB (Fig. 4). In addition, V-ATPase functions as a proton pump in yeast or other cells, and the V_0_ domain is synthesized in ER. Since both V_1_ and V_0_ subunits are present in AB proteomic data, they may be bound together, although the function and location are not still clear.

The gene expression level of hypothetical protein EMIHUDRAFT_465,517 has not been reported previously. Our data showed that the expression of the gene was up-regulated about 3-folds when *T. lutea* cells were transferred from N-sufficient to N-deficient medium. In haptophyte, there are alkenone producer and non-producer, and this protein is only found in alkenone producer, but not in non-producer. Therefore the gene might be participated in alkenone biosynthesis. Further study is required to elucidate its function.

For cycloartenol-c-24-methyltransferase, its expression level was up-regulated 12–16 folds immediately after the transfer of cells to nitrogen depletion conditions. Since about 9% of the total lipids in LB are phytosterols and cycloartenol-c-24-methyltransferase (c24) is one of the key enzyme controlling the flux of carbon into sterol biosynthesis [30], the up-regulation of gene expression is likely an indication that this gene is involved in AB development, although exact sterol biosynthesis pathway in *T. lutea* is unclear yet.

The gene annotated as coccolith scale associated protein-1 gene (Cocco) was the most up-regulated, whose expression increased 450–650 folds during the N-sufficient to N-deprived transition. Interestingly, similar up-regulation of over 500-folds of the gene was also reported in the diatom *Phaeodactylum tricornutum* in N-deprived conditions although diatoms produce triacylglycerol and do not produce coccolith or alkenones, as storage lipid [31]. In our study, the expression of the COCCO gene continued to increase during the late-exponential growth phase when the cell growth was already restricted due to nutrition deficiency, suggesting that this protein might be involved in carbon homeostasis, although it is not clear which step the protein is involved in. Study of alkenone-producing haptophyte *E. huxleyi* [32] shows that the main carbon storage in *E. huxleyi* is alkenones, membrane lipids and acid polysaccharides, not neutral polysaccharides, which is involved in biomineralization process to produce calcium carbonate crystals as cell covering [32]. In addition, results from non-aqueous phase fractionation of organelles showed that alkenones were fractionated into coccolith-producing vesicles [22]. Therefore, the coccolith scale associated protein-1 may be not associated with alkenone production.

Since SPFH domain-containing protein was isolated from AB, it might have been brought from ER during AB isolation, suggesting that it is originally located on ER membrane.

The expression of all these genes in Fig. 3, except SPFH domain-containing protein, showed a clear increase immediately after the transfer of cells to nitrogen depletion and then a decrease trend during long-term nitrogen depletion conditions (after day 16) except heat shock protein (HSP). As a consequence, the protein levels may have similar change. For example, in a previous study [27], N-deprived *T. lutea* cells induced more proteins such as ATP synthase than the cells under nutrient-enriched conditions. This increase may be due to stress-induced activation of protective mechanism under N-deprivation conditions. Meanwhile, it is worth noting that some of the transcripts such as Cocco, V-ATPase domain V_A_ and V_d_ began to increase even before the cells were transferred to N-deficient medium, suggesting that there are other mechanism regulating their expression.

Our previous work on neutral lipids from the lipid droplets of *T. lutea* showed that *T. lutea* has alkenone-containing lipid droplets [23]. So far, only TAG is known to present in eukaryotic LB, implying that AB may have alternative metabolic pathways other than TAG metabolism in other microalgae or higher plants. Polar lipids include phospholipids, hemolytic lipids and sphingomyelin, which are components of cellular membrane. Due to limited amounts, polar lipids in *T. lutea* was not studied in the previous work [23]. However, dynamic simulation studies on oil production in *Nannochloropsis* have shown that polar lipids from broken cell inner membrane due to conditions such as nitrogen deficiency can be used in TAG synthesis. With reference to our early study [23], it is likely that the synthesis of alkenone in *T. lutea* is also likely to be related to polar lipids.

With the growth of cells, the content of alkenone gradually increased, especially in logarithmic growth phase, when alkenone content also increased logarithmically. In contrast to our expectation, the alkenone content in stationary phase, especially after the cells were transferred to nitrogen-deficient medium, did not increase as sharply as the AB volume did, instead, it increased slowly or even leveled off, meaning that the accumulation of alkenone relies on cell number. On other hand, for TAG LB, oil is mainly accumulated during stationary phase. The increased AB size might be not only due to alkenone accumulation, but also fusion of small ABs although no such fusion was observed under microscope. Another possibility is that alkenone, as an energy source, is accumulated and consumed simultaneously during cell growth, leading to the slowdown of accumulation. FAMEs in neutral lipids steadily increased before the cells were transferred to nitrogen deficient-medium when alkenone content was about two times that of FAMEs, and decreased sharply after the cells were transferred to nitrogen deficient-medium when alkenone content was about seven times that of FAMEs. Since the accumulation of alkenone before the cells were exposed to nitrogen deficient-medium was almost constant, it is likely that FAMEs may be decomposed into a source of cell energy after the cells are nutritionally stressed, although alkenone is likely to be partially decomposed as well. Since FAMEs are decomposed for energy, the decomposition of alkenone is less in nutritionally sufficient condition, resulting in unchanged AB content, which is in line with the stable expression of the genes of the major proteins in lipid synthesis in our study.

Sequencing of the *T. lutea* genome has just been completed [33]. In recent studies [34, 35], the carbon flow in the *T. lutea* relatives is addressed. However, the specific pathway for the formation of alkenone, as well as functions of lipoproteins are still unclear. In this paper, the expression of lipoprotein genes is profiled in connection with change in lipuid content. We also predict the cellular location and function of these proteins. These data would help better understand alkenone metabolism pathway in *T. lutea.*

## Conclusions

Expression levels of five out of the six genes coding for AB-associated proteins are up-regulated during alkenone biosynthesis, suggesting that these genes are involved in alkenone biosynthesis in *T. lutea*. These data would help better understand alkenone metabolism and engineer for improved biofuel production in *T. lutea.*

## Methods

### Alga and culture

The haptophyte alga *Isochrysis* aff. *galbana* (Clone Tahiti), which was recently renamed as *Tisochrysis lutea* [6], was obtained from the UTEX Culture Collection of Algae at the University of Texas at Austin (UTEX LB 2307, https://utex.org/). They were cultivated in three 1.5 L culture bottles according to previous work [23]. They were subjected to nitrogen depletion to induce AB formation [23].

### BODIPY staining

BODIPY (493/503, Life Technologies, CA, USA) stock solution (1 mg/mL) was prepared using dimethyl sulfoxide. Algal culture or isolated ABs were stained with BODIPY as described [23] and examined under a fluorescent microscope (BX50, Olympus, Japan).

### Real time-qPCR

Cells were harvested by centrifugation at 8000×g for 5 min at 4 °C. The total RNAs were isolated using the TRIzol Max Bacterial RNA Isolation Kit (Life Technologies, CA, USA) according to the manufacturer’s protocol. RNA was checked for quality by gel electrophoresis on 1% agarose (w:v) non-denaturing gels and quantified using a Nanodrop spectrophotometer (SCRUM, USA). Total RNA content was adjusted to a final concentration of 1 ng/μL before PCR, and the reverse transcription was carried out using PrimeScript RT Reagent Kit (Perfect Real Time) (Takara Bio, Ohtsu, Japan) to obtain cDNAs. Primers and housekeeping gene (heat shock protein) are shown in Table 1. qPCR was conducted using SYBR Premix Ex Taq (Perfect Real Time) (Takara Bio) and analyzed by PikoReal Real-Time PCR system (Thermo Fisher Scientific, Waltham, MA) as described in a previous work [36]. Each sample was run in five parallels. The dissociation curves showed a single amplification product without primer dimer. For each primer pairs, the amplification efficiency (E) was determined by using five times dilution series starting from 100 ng cDNA to 0.16 ng cDNA to check primer specificity. The reaction efficiencies were between 90 and 110% for all the primer pairs.

**Table 1:** Primers designed for qPCR

V-ATPase subunit V1	
IsoRT-V_A-F: TCGGTCAACTGGCTCATCTC	
IsoRT-V_A-R: TGGGCGGAAACTCAGGGAA	
Hypo	
IsoRT-465,517-F: GTCACTGCCATCACCGTC	
IsoRT-465517-R: GGCTTCCTTTGTATCCTCGC	
SFPH	
IsoRT-SFPH-F: ACCATCCTCGCCTCTCCCATC	
IsoRT-SFPH-R: CGCTCTCACCCTCGTCAACC	
c24	
IsoRT-MT-c24-F: ACCATCCTCACTCCATCCTTC	
IsoRT-MT-c24-R: CCTCAACATCTCTCGCACCT	
cocco	
IsoRT-CoccoRP-F: GGCTCCAACCCTGACGACT	
IsoRT-CoccoRP-R: GACGGACAGCACCATACCCT	
V-ATPase V0 domain	
IsoRT-V_d-F: GGACGGTGGTTACTTGGAGGG	
IsoRT-V_d-R: AATGCGAGAGTTGCGGTGGA	
Heat shock (housekeeping)	
IsoRT-HSP70-F: GCTCCACTCGCATTCCCAAG	
IsoRT-HSP70-R: GTCTCCTCGCCACCCTCAC	

### Analysis of alkenone and fatty acid methyl ester (FAME)

The extraction of alkenone was performed by following a previous protocol [37]. Extracted neutral lipids and methyl-esterified polar lipids were detected as previously reported [23] using an FID-equipped capillary gas chromatograph (GC-2014AFSC; Shimadzu, Kyoto, Japan) with a CP-SIL 5CB column.
